# Antioxidant Activity of Leaf Extracts from Different *Hibiscus sabdariffa* Accessions and Simultaneous Determination Five Major Antioxidant Compounds by LC-Q-TOF-MS

**DOI:** 10.3390/molecules191221226

**Published:** 2014-12-17

**Authors:** Jin Wang, Xianshuang Cao, Hao Jiang, Yadong Qi, Kit L. Chin, Yongde Yue

**Affiliations:** 1 SFA Key Laboratory of Bamboo and Rattan Science and Technology, International Centre for Bamboo and Rattan, No. 8 Futong Dongdajie, Wangjing, Chaoyang District, Beijing 100102, China; E-Mails: wangjin@icbr.ac.cn (J.W.); caoxianshuang123@163.com (X.C.); 15956907446@163.com (H.J.); 2 Southern University Agricultural Research and Extension Center, Baton Rouge, LA 70813, USA; E-Mail: kit_chin@suagcenter.com; 3 Urban Forestry Program, College of Science and Agriculture, Southern University, Baton Rouge, LA 70813, USA

**Keywords:** *Hibiscus sabdariffa*, antioxidant activity, LC-Q-TOF-MS, method validation, DPPH, antioxidant compounds, quantitative analysis

## Abstract

*Hibiscus sabdariffa* has gained attention for its antioxidant activity. There are many accessions of *H. sabdariffa* in the world. However, information on the quantification of antioxidant compounds in different accessions is rather limited. In this paper, a liquid chromatography/quadrupole-time-of-flight mass spectrometry (LC-Q-TOF-MS) method for simultaneous determination of five antioxidant compounds (neochlorogenic acid, chlorogenic acid, cryptochlorogenic acid, rutin, and isoquercitrin) in *H. sabdariffa* leaves was developed. The method was validated for linearity, sensitivity, precision, repeatability and accuracy. The validated method has been successfully applied for determination of the five analytes in eight accessions of *H. sabdariffa*. The eight accessions of *H. sabdariffa* were evaluated for their antioxidant activities by DPPH free radical scavenging assay. The investigated accessions of *H. sabdariffa* were rich in rutin and exhibited strong antioxidant activity. The two accessions showing the highest antioxidant activities were from Cuba (No. 2) and Taiwan (No. 5). The results indicated that *H. sabdariffa* leaves could be considered as a potential antioxidant source for the food industry. The developed LC-Q-TOF-MS method is helpful for quality control of *H. sabdariffa*.

## 1. Introduction

*Hibiscus sabdariffa* L. (family: Malvacea) is an annual herb shrub popularly known as Roselle or Sorrel [1]. The plant is commonly used as beverages and folk medicines [2]. Many *H. sabdariffa* accessions (samples of a crop variety collected at a specific location and time) are widely cultivated in tropical and subtropical countries. In Africa, the leaves of *H. sabdariffa* are usually consumed as vegetables in the preparation of soups and sauces [3]. The leaf extract has been found to possess many bioactive properties, such as anti-oxidant [1,4], anti-tumor [5], anti-hyperammonemic [4], anti-atherosclerotic [3], anti-filarial [6] and anti-hyperlipidemic activities [1,7]. The leaves of *H. sabdariffa* are rich in phenolics, which could be responsible for the antioxidant capacity. Yields of *H. sabdariffa* leaves may be about 10 t/ha. Unfortunately, only the calyces of *H. sabdariffa* are used widely and the leaves are usually ignored and discarded in most countries [3].

There has been growing interest in natural antioxidants found in plants because of the carcinogenic effects of synthetic antioxidants [8]. Oxidative stress causes collapse of the mitochondrial membrane potential, which is associated with many age-related diseases [9,10]. Dietary antioxidants, such as vitamin C and phenolic compounds, present in foods contribute to defense against oxidative stress [11,12]. Overall, natural antioxidants can protect the human body from free radicals and retard the lipid oxidative rancidity in foods. According to the previous studies, *H. sabdariffa* has long been recognized as a source of antioxidants [1,4,13]. However, the *H. sabdariffa* leaves from different countries could have different chemical compositions, which may influence their antioxidant activity. Our research group has identified five major antioxidant compounds (including neochlorogenic acid, chlorogenic acid, cryptochlorogenic acid, rutin, and isoquercitrin) from *H. sabdariffa* leaves. To the best of our knowledge, there is still no reported work on the comparison of the contents of the main antioxidant compounds in the leaves of *H. sabdariffa* from different accessions. 

Time-of-flight tandem mass spectrometry (TOF-MS) is a powerful technology, which has been successfully applied for qualitative and quantitative analysis of the chemical constituents in medicinal plant [14,15]. The aim of this study was to develop and validate a LC-Q-TOF-MS method for determination of the contents of the five antioxidant compounds in the different *H. sabdariffa* accessions. In addition, total antioxidant capacity of different accessions of *H. sabdariffa* was evaluated by using DPPH free radical scavenging assay. This study is helpful for the full utilization of *H. sabdariffa*.

## 2. Results and Discussion

### 2.1. Optimization of LC-Q-TOF-MS Conditions

**Figure 1:**
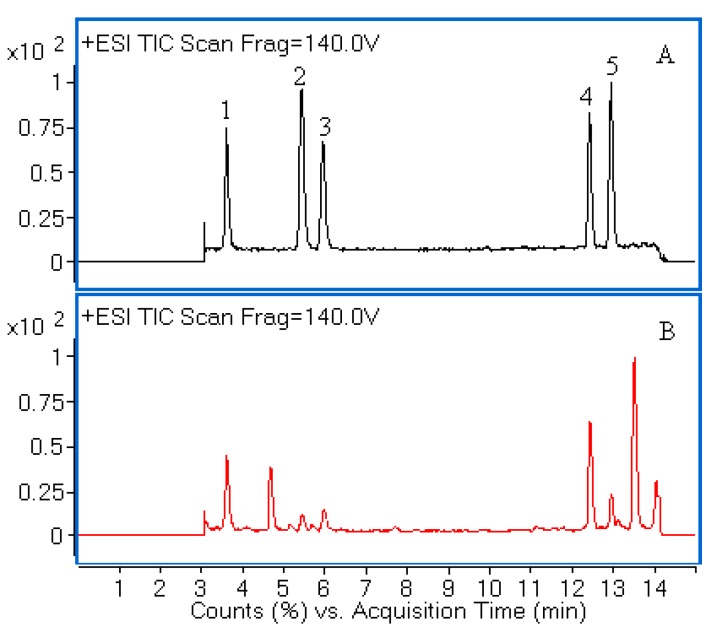
Liquid chromatography/quadrupole-time-of-flight mass spectrometry (LC-Q-TOF-MS) total ion chromatograms (TIC) of a mixture of five standards (**A**) and the extract of *H. sabdariffa* (**B**). Peaks **1**, **2**, **3**, **4** and **5** correspond to neochlorogenic acid, chlorogenic acid, cryptochlorogenic acid, rutin and isoquercitrin, respectively.

A series of preliminary experiments were carried out in order to optimize the LC-Q-TOF-MS conditions. After comparison with YMC-UltraHT pro C_18_ (100 mm × 2.0 mm i.d., 2 μm) and Agilent Eclipse XDB C_18_ (150 mm × 2.1 mm i.d., 3.5 μm) column, Agilent Eclipse Plus C_18_ (150 mm × 2.1 mm i.d., 1.8 μm) column gave a better separation of the analytes within 15 min. Different mobile phase compositions such as acetonitrile-water and aqueous acetonitrile-acid solvents were tested. The acidified mobile phase (0.1% formic acid) and gradient mode were necessary to achieve a satisfactory MS response and chromatographic separation. To obtain more stable product ions and high responses, MS parameters including fragmentor voltage and drying gas temperature were optimized. Moreover, the positive mode was selected for MS analysis as it had better sensitivity of ion response than that of negative mode. The typical total ion current (TIC) chromatograms were shown in Figure 1. The chemical structures of five compounds were characterized by comparing accurate mass and their retention times with those of standard compounds. 

### 2.2. Method Validation

The regression equations, linear ranges, LODs and LOQs values of five analytes were performed using the developed LC-Q-TOF-MS method. As shown in Table 1, the calibration curves of the proposed method were generated by using polynomial regression. Reasonable correlation coefficient values (R^2^ ≥ 0.9993) indicated good correlations between the concentrations of five analytes and their peak areas within the tested ranges. In this study, LOD and LOQ values were less than 0.09 µg/mL and 0.19 µg/mL, respectively, which were small enough to meet the need for determination of the analytes in *H. sabdariffa*.

**Table 1:** Calibration curves, linearity, limits of detection (LOD) and limits of quantification (LOQ) of the five analytes.

Analytes	Regression Equation	Linear Range (µg/mL)	Correlation Coefficient (R^2^)	LOD (µg/mL)	LOQ (µg/mL)
Neochlorogenic acid	y = −833.90·x^2^ + 139143.92·x + 6461.02	0.75–48.00	0.9997	0.05	0.19
Chlorogenic acid	y = −1795.39·x^2^ + 239416.18·x − 4374.15	0.19–48.00	0.9993	0.05	0.19
Cryptochlorogenic acid	y = −677.23·x^2^ + 119252.57·x + 2703.14	0.19–48.00	0.9998	0.09	0.19
Rutin	y = −1265.45·x^2^ + 220749.21·x + 1297.41	0.09–48.00	0.9999	0.02	0.09
Isoquercitrin	y = −1970.01·x^2^ + 256239.13·x + 3378.03	0.09–48.00	0.9997	0.02	0.09

As shown in Table 2, the repeatability present as RSD (*n* = 6) was between 2.77% and 4.89% of the five investigated compounds. The overall intra- and inter-day variations (RSD) of five analytes for peak areas were in the range from 1.80% to 3.10%, and 1.05% to 3.93%, respectively. Meanwhile, the retention time variations (RSD) were less than 0.21% and 0.52%, respectively (Table 2). 

**Table 2:** Repeatability and precision of the investigated analytes.

Analytes	Repeatability (RSD, *n* = 6) %	Intra-day		Inter-day	
		(RSD, *n* = 6) %		(RSD, *n* = 6) %	
		Retention Time	Peak Area	Retention Time	Peak Area
Neochlorogenic acid	3.92	0.09	2.04	0.52	3.91
Chlorogenic acid	4.89	0.21	2.54	0.46	1.05
Cryptochlorogenic acid	4.47	0.15	1.84	0.47	1.29
Rutin	2.77	0.09	1.80	0.22	2.96
Isoquercitrin	2.81	0.06	3.10	0.19	3.93

The average recoveries obtained in this study ranged from 86.28% to 101.26%, while RSD were all less than 4.88% (Table 3). Therefore, the proposed LC-Q-TOF-MS method was sensitive, precise, and accurate for quantitative evaluation of the five analytes in *H. sabdariffa.*

**Table 3:** Recovery test of the five compounds in *H. sabdariffa* leaves (*n* = 3).

Analytes	Original Amount (µg)	Added (µg)	Found (µg)	Recovery (%)	RSD (%)
Neochlorogenic acid	594.63	412.50	1007.11	99.99	3.88
	594.63	825.00	1317.16	87.58	1.38
Chlorogenic acid	83.37	45.00	122.13	86.28	1.95
	83.37	90.00	166.31	92.23	4.32
Cryptochlorogenic acid	166.01	100.00	262.57	96.56	4.76
	166.01	200.00	355.90	94.94	3.13
Rutin	726.71	562.50	1296.29	101.26	4.11
	726.71	1125.00	1793.28	94.81	4.03
Isoquercitrin	147.97	112.50	259.52	99.15	4.88
	147.97	225.00	345.92	87.98	0.83

### 2.3. Quantitative Analysis

**Figure 2:**
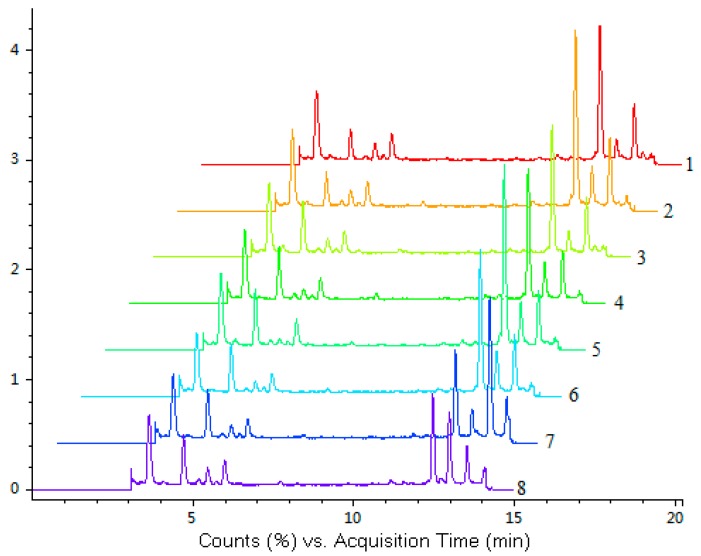
LC-Q-TOF-MS total ion chromatograms (TIC) of eight samples of *H. sabdariffa*. Different samples (No. 1–8) are listed in Table 4.

The developed LC-Q-TOF-MS method was subsequently applied to simultaneous quantification of the five investigated compounds in eight accessions of *H. sabdariffa.* The fingerprint chromatograms of eight samples of *H. sabdariffa* are shown in Figure 2.

**Table 4:** Investigated leaves of eight different accessions of *H. sabdariffa*. The plant identification (PI) numbers of accession label were assigned by the United States Department of Agriculture-Agricultural Research Service (USDA-ARS).

No.	Country (Seed Source)	Accession Label
1	India	PI-180026
2	Cuba	PI-207920
3	Sudan	PI-267778
4	Nigeria	PI-268100
5	Taiwan	PI-273388
6	Nigeria	PI-274245
7	Senegal	PI-275413
8	Sudan	PI-496938

The identification of the investigated compounds was performed by comparison of their retention time and accurate MS with those of reference standards. The molecular ion peaks and the MS data were shown in Table 5.

**Table 5:** MS data of the five compounds from *H. sabdariffa* by LC-Q-TOF-MS.

No.	RT (min)	[M+H]^+^	Main Fragments	Calculated *m/z*	Error		Formula	Identification
					mDa	ppm		
1.	3.62	355.1023	163.0390	355.1024	0.10	0.21	C_16_H_18_O_9_	Neochlorogenic acid
2.	5.44	355.1026	163.0391	355.1024	−0.65	−0.20	C_16_H_18_O_9_	Chlorogenic acid
3.	5.96	355.1032	163.0395	355.1024	−0.20	−0.48	C_16_H_18_O_9_	Cryptochlorogenic acid
4.	12.42	611.1612	465.1025	611.1607	−0.60	−0.91	C_27_H_30_O_16_	Rutin
5.	12.95	465.1029	303.0500	465.1028	−0.10	−0.31	C_21_H_20_O_12_	Isoquercitrin

The contents of five analytes in *H. sabdariffa* are presented in Table 6. The results showed that the content of each analyte varied greatly among different accessions of *H. sabdariffa.* The variations (RSD) in the content of five analytes were in the range from 10.5% to 54.9%, which could lead to the variation of antioxidant effects. Among the tested samples, the sample No. 5 from Taiwan had the highest contents of the five analytes, while the sample No. 7 from Senegal had the lowest amount. There was no significant difference in the content between the sample No. 5 and sample No. 2. Therefore, the two accessions of *H. sabdariffa* from Cuba and Taiwan had higher contents of five marker compounds compared to the other tested samples.

**Table 6:** Contents (µg/g) of five antioxidant compounds in different *H. sabdariffa* leaves (*n* = 3). Sample No. corresponds to Table 4. Relative standard deviation (RSD) were obtained based on the contents of eight samples. Total—the sum of the five investigated compounds. a, b, c, d, e—the same letter in column indicates homogeneous groups obtained by the ANOVA (*p* = 0.05).

No.	Contents (µg/g) (Mean ± SD)					
	Neochlorogenic Acid	Chlorogenic Acid	Cryptochlorogenic Acid	Rutin	Isoquercitrin	Total
1	6875.0 ± 251.6	975.0 ± 27.2	2318.8 ± 79.8	12860.4 ± 258.5	966.7 ± 28.9	23995.9 bc
2	7633.3 ± 41.6	993.8 ± 43.8	2181.3 ± 18.8	19356.3 ± 409.7	2270.8 ± 78.2	32435.5 a
3	6577.1 ± 50.1	879.2 ± 9.6	1991.7 ± 73.2	12556.3 ± 151.7	1029.2 ± 23.7	23033.5 cd
4	6912.5 ± 136.2	645.8 ± 20.1	1952.1 ± 68.6	12560.4 ± 534.4	1998.0 ± 78.8	24068.8 bc
5	7512.5 ± 304.1	350.0 ± 16.5	2356.3 ± 109.5	20472.9 ± 653.0	2518.8 ± 109.5	33210.5 a
6	5545.8 ± 103.9	677.1 ± 26.0	1635.4 ± 68.6	14058.3 ± 237.4	2341.7 ± 23.7	24258.3 b
7	5979.2 ± 191.4	800.0 ± 38.0	1637.5 ± 65.9	7245.8 ± 196.1	1466.7 ± 34.4	17129.2 e
8	6841.7 ± 469.1	1052.1 ± 65.7	2202.1 ± 78.2	7845.8 ± 219.6	4702.1 ± 78.6	22643.8 d
RSD %	10.5	29.2	13.9	35.4	54.9	

### 2.4. Antioxidant Activity

All the samples were evaluated for their antioxidant activities by DPPH free radical scavengingassay. Bamboo leaves were used as a comparison. As shown in Table 7, there was a good linear relationship (R^2^ ≥ 0.9897) between the free radical scavenging rate and the final concentration of *H. sabdariffa* leaves in the ranges 37.33–320.00 µg/mL (dry weight basis for leaf). The IC_50_ value was used as a significant indicator of antioxidant ability. Moreover, the lower the IC_50_ value, the higher the antioxidant activity of samples. In terms of IC_50_ values, the capacity of DPPH radical scavenging activity was in a decreasing order: 2 > 5 > 1 > 6 > 4 > 3 > 8 > 7 (Table 7). According to the Section 3.1, all the accessions had grown under the same conditions, such as the cultivated soil, cultivated method and local climate. The leaves of *H. sabdariffa* were collected at the same time. Therefore, the different antioxidant activity of *H. sabdariffa* leaves may be due to the differences among these accessions. Furthermore, the differences in the antioxidant activity can be explained by the differences of the contents of antioxidant compounds. 

As seen from Table 6 to Table 7, the content of rutin was higher than that of other investigated compounds. Meanwhile, rutin exhibited strong DPPH radical scavenging activity and its IC_50_ value was 4.54 ug/mL. Rutin is one of the most important dietary flavonoid that is widely consumed from plant-derived foods. Rutin has significant pharmacological activities, such as antioxidation and health benefits. Over 130 registered therapeutic medicinal preparations are containing rutin in their formulations [16]. The strong antioxidant ability of rutin has been proven by several studies, especially for free radical scavenging activity [17,18,19]. Therefore, the high antioxidant activity of the leaves of *H. sabdariffa* was associated with increased concentrations of the investigated compounds. In most of the tested samples, the five investigated analytes were considered as the major antioxidant compounds. Besides these five compounds, there are also several unknown compounds that possess antioxidant activity in *H. sabdariffa* [20]*.*

**Table 7:** Antioxidant activity of the eight accessions of *H. sabdariffa.* Sample No. corresponds to Table 4.

Samples	Regression Equation	R^2^	IC_50_ (µg/mL, Dry Weight Basis for Leaf)
1	y = 0.2776·x + 5.1522	0.9932	161.91
2	y = 0.2767·x + 7.3187	0.9897	154.65
3	y = 0.2190·x + 5.1677	0.9945	204.72
4	y = 0.2092·x + 8.1093	0.9937	200.29
5	y = 0.2730·x + 6.9611	0.9931	157.65
6	y = 0.2117·x + 9.2249	0.9944	192.61
7	y = 0.1818·x + 5.2144	0.9987	247.44
8	y = 0.1729·x + 14.2790	0.9935	207.73
Bamboo leaves	y = 0.2497·x + 0.7810	0.9908	197.67
Rutin	y = 10.492·x + 2.3650	0.9941	4.54
Neochlorogenic acid	y = 9.9885·x - 0.2967	0.9992	5.04
Chlorogenic acid	y = 9.6473·x + 1.5983	0.9982	5.02
Cryptochlorogenic acid	y = 9.5822·x − 0.4380	0.9980	5.26
Isoquercitrin	y = 12.059·x + 7.5899	0.9947	3.52

As to antioxidant activity of *H. sabdariffa*, several studies have focus on its flower [21,22]. In contrast with the leaves, the calyces of *H. sabdariffa* are rich in hydroxycitric acid and hibiscus acid [23]. Previous studies have indicated that both the total content of flavonoid and radical scavenging activity of *H. sabdariffa* leaves were higher than those of *H. sabdariffa* flowers [3,13]. Furthermore, the *H. sabdariffa* leaf extracts possessed stronger antioxidant capacity than mulberry leaf extracts, which are rich in polyphenolic compounds [3,24]. 

In China, antioxidant of bamboo leaves (AOB) has been listed in the national standards (standard No.GB2760) in 2004. AOB as a kind of food antioxidant has been used in edible oil, meat product, aquatic product and puffed food as a kind of natural antioxidant [25]. In our previous study, the methanolic extract of *Bambusa textilis* possessed the highest antioxidant activity among the selected bamboo species [26]. As seen from Table 7, the IC_50_ value of bamboo leaves (*B. textilis*) was 197.67 µg/mL, which was similar with the IC_50_ values of *H. sabdariffa* leaves. These results indicated that *H. sabdariffa* leaves could be considered as a potential antioxidant source for the food industry. Further studies on the antioxidant mechanism and safety evaluation of *H. sabdariffa* leaves are needed [27].

## 3. Experimental

### 3.1. Chemicals and Materials

**Figure 3:**
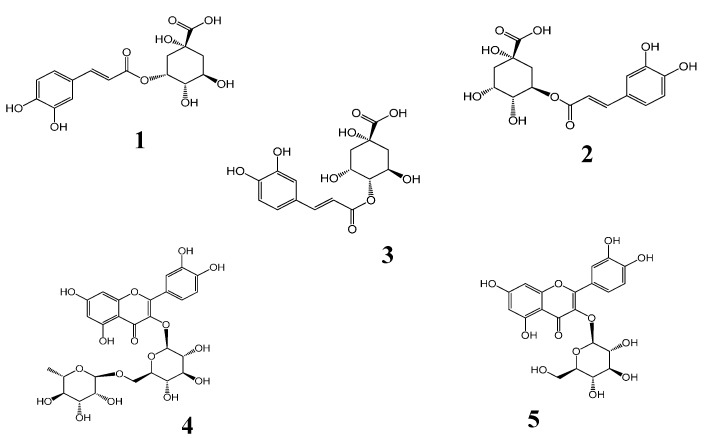
Chemical structures of the five investigated compounds including neochlorogenic acid (**1**), chlorogenic acid (**2**), cryptochlorogenic acid (**3**), rutin (**4**) and isoquercitrin (**5**).

HPLC grade acetonitrile, methanol and formic acid were purchased from Fisher Scientific (Fair Lawn, NJ, USA). Ultrapure water was obtained from a Pall purification system (Purelab Plus, Pall, Port Washington, NY, USA). 2, 2-diphenyl-1-picrylhydrazyl (DPPH) was purchased from Sigma-Aldrich (St. Louis, MO, USA). All other analytical-grade chemicals were purchased from Beijing Chemical Works (Beijing, China). Neochlorogenic acid (**1**), chlorogenic acid (**2**), cryptochlorogenic acid (**3**), and isoquercitrin (**5**) standards were purchased from Beijing Equation Biotechnology Co., Ltd. (Beijing, China). The standards of rutin (**4**) were purchased from Acros Organics (Morris Plains, NJ, USA). Their purities were above 97%. Chemical structures of the five investigated compounds were shown in Figure 3. 

The bamboo leaves were collected from Nanchang, Jiangxi Province, China and authenticated by Jiusheng Peng from Jiangxi Academy of Forestry. Bamboo leaves were dried in the shade, ground to powder, and stored at −20 °C. All the tested *H. sabdariffa* accessions were obtained from United States Department of Agriculture-Agricultural Research Service (USDA-ARS) Plant Genetic Resources Conservation Unit in Griffin, Georgia. Then, the seeds of all the accessions were planted in a greenhouse in Southern University Horticultural Farm at the same time on 27 March 2012. The leaf samples of all accessions were collected from top 5th, 6th, and 7th leaves on 27 June 2012. The leaves of *H. sabdariffa* were obtained from Southern University and A & M College, Baton Rouge, LA, USA, and were identified by Kit L. Chin (Southern University Agricultural Research and Extension Center). A total of 8 accessions of *H. sabdariffa* were included in this study (Table 4).

### 3.2. Sample Preparation

Dried leaf powder (0.20 g) was accurately weighted and placed in a 40 mL amber glass bottle with polytetrafluoroethylene (PTFE) screw cap. After adding 25 mL of 70% (*v*/*v*) methanol to the glass bottle, the mixture was extracted in an ultrasonic cleaning bath (KQ-500, 500 W, Kunshan Ultrasonic Instruments Co., Ltd., Kunshan, China) for 30 min at room temperature. 1 mL of supernatant was transferred into a 5 mL volumetric flask and diluted to volume with 70% (*v*/*v*) methanol. The final solution was filtered through a syringe filter membrane (0.45 µm). The filtrate was stored at −20 °C for further analysis.

### 3.3. Chromatographic Conditions

Chromatographic analysis was carried out on an Agilent Series 1290 system (Agilent Technologies, Santa Clara, CA, USA), equipped with a diode array detector (DAD), a quaternary pump and an autosampler. Chromatographic separation was performed on an Agilent Eclipse Plus C_18_ (150 mm × 2.1 mm i.d., 1.8 μm) at 30 °C. The mixture of (A) acetonitrile and (B) 0.1% (*v*/*v*) formic acid in water was chosen as mobile phase. The gradient elution program was as follows: 0–8 min, 90%–85% B; 8–14 min, 85%–70% B; 14–15 min, 70%–10% B. The flow rate was set at 0.25 mL/min. A post-run equilibrium time of 5 min and an injection volume of 2 μL were used for all samples. 

### 3.4. Mass Spectrometry

The quantitative analysis of the five compounds was performed using Q-TOF-MS system (model 6540, Agilent Technologies, Santa Clara, CA, USA) equipped with a jet stream ESI interface. The TOF/MS system was operated in positive mode and the mass analysis conditions were set as follows: drying gas (N_2_) flow rate, 10 L/min; drying gas temperature, 350 °C; fragmentor voltage, 140 V; nebulizer, 45 psi; capillary voltage, 4000 V; skimmer voltage, 65 V; sheath gas temperature, 250 °C; nozzle voltage, 500 V; octopole RF voltage, 750 V. Mass spectra were acquired in the *m*/*z* range of 100 to 800. The MS data were collected in a MS scan mode. The molecular masses of the precursor ions were accurately detected using two reference masses (121.050873 and 922.009798). During the analysis, the reference masses were infused to calibrate the MS system. All the operations and analysis of data were controlled using MassHunter B.04.00 software (Agilent Technologies, Santa Clara, CA, USA).

### 3.5. Calibration Curves and Limits of Detection

Stock solution of the five analytes was diluted to appropriate concentrations for construction of calibration curves. The calibration curves were constructed by plotting the peak area (EIC signal of MS) *versus* the concentration of each analyte. For each analyte, at least seven concentrations of the solution were analyzed. According to ICH guideline [28], the signal-to-noise (S/N) method was selected for testing limits of detection (LOD) and limits of quantification (LOQ). A signal-to-noise ratio (S/N) of three is used for evaluating LOD and signal-to-noise ratio of ten is accepted for evaluating LOQ. The LOD and LOQ were determined by injecting a series of diluted standard solutions until the signal-to-noise ratio (S/N) for the standards reached a 3:1 ratio for LOD and 10:1 for LOQ, respectively. 

### 3.6. Accuracy, Precision and Repeatability

The spike recovery test was carried out to evaluate the accuracy of the method. Known amounts of each standard solution at low and high concentration levels were mixed with certain amount (0.10 g) of sample No. 7. Then the mixtures were extracted and analyzed using the developed method. Three replicates were prepared for each level.

Recovery (%) = (amount found − original amount)/spiked amount × 100
(1)

Intra- and inter-day variations were applied to evaluate the precision of the developed method. For intra-day precision, the solution of sample No. 7 was analyzed for six replicates within one day, while for inter-day test, the same sample was examined in duplicates for consecutive three days. The relative standard deviation (RSD) was taken as a measurement of precision. Six independent samples (No. 7) were extracted and analyzed in parallel by the developed method for the measurement of repeatability.

### 3.7. DPPH Free Radical Scavenging Activity

The free radical scavenging activity of the crude extracts was assessed by using the method described by Koolen and with a slight modification [29]. The final concentration of DPPH solution in this study was 26.67 ug/mL for 0.068 mM solution, which could give a moderate absorbance based on Beer’s law [30]. Briefly, an aliquot (1 mL) of each plant sample with different concentrations (corresponding to a 0.96 mg, 0.80 mg, 0.48 mg, 0.16 mg, 0.11 mg of dried plant leaves) was added to DPPH solution (40 ug/mL, 2 mL) in methanol. An aliquot (1 mL) of each standard compound with different concentrations (2.50 ug/mL, 5.00 ug/mL, 10.00 ug/mL, 15.00 ug/mL, 20.00 ug/mL) was added to DPPH solution (40 ug/mL, 2 mL). The mixture was incubated with vigorous shaking for 30 min at 37 °C. The absorbance was measured at 517 nm. IC_50_ value (concentration providing 50% inhibition) was calculated using a calibration curve. The percentage inhibition was measured using the following equation:

DPPH inhibition (%) = (A_control_ − A_sample_)/A_control_ × 100 (2)

where A_control_ was the absorbance of control sample and A_sample_ was the absorbance of sample with the crude extract. 

### 3.8. Statistical Aanlysis

Statistical significance was performed applying one-way ANOVA followed by Duncan’s test at *p* = 0.05, using SPSS Statistics version 17.0 (SPSS Inc., Chicago, IL, USA).

## 4. Conclusions

A powerful analytical method LC-Q-TOF-MS was developed to quantify five antioxidant compounds in eight accessions of *H. sabdariffa*. The method was validated and proven to be simple, sensitive and reliable. Comparative analysis of all samples indicated that the leaves of *H. sabdariffa* were rich in rutin and exhibited strong antioxidant activity. The contents of five antioxidant compounds in *H. sabdariffa* leaves were related with their antioxidant activity. Therefore, the five investigated compounds could be the predominant contributors to the antioxidant activity. The two accessions showing the highest antioxidant activities were from Cuba (No. 2) and Taiwan (No. 5). In addition to their traditional usage in food and medicine, the leaves of *H. sabdariffa* may be considered as a potential antioxidant source for the food industry. 
